# Advancements in the clinical management of adhesive small bowel obstruction: A perspective

**DOI:** 10.1097/MD.0000000000043246

**Published:** 2025-07-04

**Authors:** Chang-Jun Shen, Jin-Jiang Wang

**Affiliations:** a Second Ward of General Surgery Department, Yan’an People’s Hospital, Yan’an, China; b First Ward of General Surgery Department, Yan’an People’s Hospital, Yan’an, China.

**Keywords:** adhesion, adhesive small bowel obstruction, clinical management, diagnosis, pathophysiology

## Abstract

This study rigorously addresses adhesive small bowel obstruction (ASBO), a common yet severe complication following abdominal surgery, marked by fibrous adhesions that disrupt normal intestinal operations. These adhesions often result in profound symptoms including severe abdominal pain and vomiting, and may lead to critical outcomes such as intestinal ischemia and necrosis. It delineates the considerable strain ASBO places on healthcare systems globally, highlighting its frequent occurrence post-surgery and the associated high medical costs and negative impacts on patient quality of life. The study explores recent advancements in the management of ASBO, focusing on innovative diagnostic techniques, surgical procedures, and therapeutic interventions designed to decrease recurrence rates and enhance patient recovery outcomes. Significant emphasis is placed on the effectiveness of a barrier agent shown to significantly decrease the necessity for surgical reinterventions by preventing the formation of new adhesions. The study proposes a modernized management strategy for ASBO, advocating for the adoption of the latest diagnostic and treatment approaches tailored to individual patient conditions. It recommends the integration of cutting-edge technologies and personalized treatment plans, aimed at boosting treatment efficacy and patient well-being, thereby mitigating the overall healthcare burden.

## 1. Introduction

Adhesive small bowel obstruction (ASBO) represents a prevalent abdominal emergency, characterized by fibrous adhesions within the peritoneal cavity, leading to partial or complete obstruction of the small intestine.^[1]^ This condition not only emerges as a frequent postoperative complication but also constitutes a substantial cause for emergency department admissions.^[2]^ The etiology of ASBO involves intricate mechanisms, including the inflammatory response post-surgery, the tissue repair process, and changes in peritoneal reactivity, culminating in severe clinical manifestations such as intense abdominal pain, vomiting, distension, and halted bowel movements.^[3,4]^ In extreme scenarios, this can escalate to intestinal ischemia, necrosis, and potentially, patient mortality.

Epidemiologically, ASBO places a significant strain on global public health systems.^[1]^ Estimates suggest that approximately 3% to 6% of individuals undergoing abdominal surgeries may develop ASBO subsequently. Moreover, the hospitalization required for managing ASBO not only augments the financial load on healthcare infrastructures but also severely deteriorates the affected individuals’ life quality.^[5]^ The repercussions of prolonged hospital stays, recurrent surgical interventions, and associated complications profoundly impact the patients’ physical and mental well-being.

The critical nature of ASBO underscores the imperative for research focused on its clinical management’s evolution.^[6]^ Despite the foundational reliance on conservative and surgical approaches for ASBO management, the persistent challenges of recurrence and the inherent risks of treatment modalities necessitate a reevaluation.^[7]^ Thus, this investigation seeks to delineate recent advancements in the realm of ASBO treatment, spotlighting novel diagnostic techniques, therapeutic strategies, and preemptive measures aimed at augmenting treatment efficacy, curtailing complications, and minimizing recurrence rates.^[8,9]^ Such endeavors aim to mitigate the repercussions on both the individuals afflicted and the broader public health landscape. Through an analytical examination of prevailing treatment paradigms and prospective therapeutic innovations, this study endeavors to furnish clinical practitioners with informed guidance, thereby facilitating enhanced treatment outcomes and life quality for individuals grappling with ASBO.

## 2. The pathophysiology of ASBO

The etiology and impact of ASBO on patient physiology and clinical manifestations encompass intricate mechanisms ranging from cellular responses to systemic physiological reactions.

### 2.1. Mechanisms underlying adhesion formation

Adhesions predominantly arise from aberrant healing processes post-abdominal surgery, infections, inflammation, or trauma.^[1,10,11]^ This sequence initiates with peritoneal damage, triggering a fibrogenic response characterized by fibroblast proliferation and collagen deposition, culminating in the formation of fibrous tissue.^[10,11]^ These fibrous formations create adhesion bands within the abdominal cavity, constraining organ mobility. Such restrictions can lead to the small intestine being subjected to tension or torsion, precipitating an obstruction.

### 2.2. Physiological consequences of small intestinal obstruction

The development of small intestinal obstruction results in the accumulation of intraluminal contents above the obstruction site, elevating intraluminal pressure and bowel wall tension, thereby compromising bowel wall vascular circulation.^[1,11,12]^ Chronic elevated pressure may induce bowel wall edema, ischemia, and potentially necrosis. Furthermore, obstruction disrupts intestinal fluid and electrolyte homeostasis, leading to dehydration and electrolyte imbalances.^[12]^ The impaired motility in the obstructed segment, coupled with increased intraluminal pressure, may facilitate bacterial translocation and endotoxin absorption, heightening infection risks.^[12]^

### 2.3. Clinical presentation of ASBO

ASBO typically manifests with symptoms including acute abdominal pain, vomiting, abdominal distension, and either diminished or absent bowel sounds.^[13,14]^ Pain is often intermittent, initially localized to the obstruction site but may diffuse across the abdomen over time. Obstruction impedes the passage of intestinal contents, prompting episodes of vomiting whose characteristics are influenced by the obstruction’s location and severity.^[13,14]^ Abdominal distension results from the upstream accumulation of intestinal contents and gas. Progression of the condition may lead to dehydration and electrolyte imbalance signs, such as thirst, fatigue, and tachycardia.

A comprehensive understanding of ASBO’s pathophysiology and clinical presentations is pivotal for prompt diagnosis and effective management.^[13,14]^ Accurate identification of these clinical signs enables healthcare providers to devise optimal treatment strategies, mitigating the risk of complications and enhancing patient quality of life.

## 3. Diagnostic approaches for ASBO

Diagnosing ASBO involves a systematic process that incorporates detailed clinical evaluation, meticulous history gathering, and the employment of sophisticated imaging techniques. Establishing an accurate diagnosis is essential for formulating an efficacious therapeutic strategy.

### 3.1. Clinical evaluation and history collection

The initial step in diagnosing ASBO is a comprehensive clinical assessment.^[14–17]^ Physicians conduct a thorough inquiry into the patient’s presenting symptoms, including but not limited to abdominal pain, vomiting, distension, and alterations in defecation and flatulence.^[14–17]^ The collection of the patient’s medical history is pivotal, with a focus on prior abdominal surgeries, inflammatory conditions, trauma, or any incidents that might precipitate adhesion development.^[14–17]^ Physical examination is critical, with emphasis placed on assessing abdominal distension, pinpointing areas of tenderness, and evaluating bowel sounds.^[14]^

### 3.2. Imaging diagnostic techniques

Imaging studies are indispensable in the diagnostic algorithm of ASBO, with several modalities playing critical roles^[14,18–20]^:

#### 3.2.1. X-ray

Abdominal X-rays are instrumental in identifying signs of gas accumulation and bowel lumen dilation, offering a preliminary diagnostic insight into ASBO.^[14]^

#### 3.2.2. Ultrasound

Despite its limited sensitivity to intraperitoneal gas, ultrasound is valuable for delineating the obstruction’s exact location and detecting fluid accumulation.^[18,19]^

#### 3.2.3. Computed tomography

CT scanning stands as a preferred diagnostic tool, providing comprehensive insights into the intestines, elucidating obstruction location, adhesion extent, and potential bowel wall ischemia or necrosis.^[18–20]^

#### 3.2.4. Magnetic resonance imaging

Magnetic resonance imaging excels in soft tissue evaluation and is particularly advantageous for ambiguous cases or scenarios where minimizing radiation exposure is paramount, such as during pregnancy.^[14]^

### 3.3. Diagnostic criteria and differential diagnosis

A confirmed ASBO diagnosis necessitates synthesizing clinical symptoms, physical examination outcomes, and imaging findings.^[1,7,14]^ The diagnostic criteria essentially combine typical clinical symptoms with corroborative imaging evidence.^[7,14]^ Differential diagnosis is crucial to exclude conditions with similar symptomatology, including acute appendicitis, alternative etiologies of intestinal obstruction (e.g., tumors, Crohn disease), and nonsurgical acute abdominal conditions (e.g., pancreatitis).^[7,14]^

This methodical and comprehensive diagnostic approach enables physicians to accurately identify ASBO, facilitating the development of a tailored treatment plan aimed at reducing complications and optimizing patient outcomes.

## 4. Clinical managements of ASBO

The management of ASBO encompasses 2 primary therapeutic approaches: conservative and surgical intervention^[21–41]^ (Table 1). The selection of a suitable treatment strategy hinges on an array of factors, including the severity of the obstruction, the overall health status of the patient, and the presence or absence of complicating factors.

**Table 1:** Clinical managements in patients with ASBO of clinical studies.

Patients with	Type of study	Treatment	Key findings	Reference
Patients with ASBO	Case report	Parenteral nutrition	Successful management of condition	Suminaga et al 2022^[21]^
Patients with ASBO	Randomized controlled trial	Laser acupuncture	Not reported	Shih et al 2021^[22]^
Patients with ASBO	Randomized controlled trial	Transnasal ileus versus nasogastric tube	Endoscopic placement of the ileus tube for ASBO is convenient and beneficial, despite some risks	Chen et al 2012^[23]^
Patients with ASBO	Randomized controlled trial	Magnesium oxide and simethicone	Effectively hastened the resolution of partial ASBO and shortened hospital stays	Chen et al 2005^[24]^
Patients with ASBO	Randomized controlled trial	Short versus long tubes	Tube decompression can be safely administered to patients with ASBO upon hospital admission	Fleshner et al 1995^[25]^
Patients with ASBO	Observational study	Gastrografin	Safe and useful for conservative management	Miquel et al 2017^[26]^
Patients with ASBO	Retrospective study	Sesame oil	Sesame oil was a safe and effective adjunct to the standard treatment of partial ASBO.	Ji et al 2010^[27]^
Patients with ASBO	Randomized controlled trial	Gastrografin	Gastrografin safely aids in managing ASBO by reducing surgery rates, resolution time, and hospital stays	Farid et al 2010^[28]^
Patients with ASBO	Randomized clinical trial	Octreotide and methylglucamine diatrizoate	Combined octreotide and methylglucamine diatrizoate safely accelerate ASBO resolution in older adults through a specific therapeutic effect	Zhang et al 2006^[29]^
Patients with ASBO	Observational study	Gastrografin	Gastrografin is safe for treating ASBO and can reduce surgical needs when conservative approaches fail	Choi et al 2002^[30]^
Patients with ASBO	Observational study	Gastrografin	Gastrografin use in ASBO after failed conservative treatment is safe and decreases surgical interventions	Choi et al 2005^[31]^
Patients with ASBO	Randomized clinical trial	Nasogastric tube with Gastrografin	Gastrografin via nasogastric tube and subsequent long tube (LT) may be effective for ASBO, matching the long-term efficacy of initial LT	Nishie et al 2022^[32]^
Patients with ASBO	Randomized controlled trial	Gastrografin	Gastrografin accelerates resolution of ASBO by a specific therapeutic effect	Burge et al 2005^[33]^
Patients with ASBO	Randomized clinical trial	Gastrografin	Gastrografin administration is of no benefit in patients with ASBO	Scotté et al 2017^[34]^
Patients with ASBO	Randomized clinical study	Gastrografin	Oral Gastrografin helps manage ASBO and shortens hospital stays	Biondo et al 2003^[35]^
Patients with ASBO	Clinical study	Telebrix Gastro	Significantly aid in managing patients suspected of having a ASBO	Aulin et al 2005^[36]^
Patients with ASBO	Retrospective study	Medication	Not Reported	Sazhin et al 2018^[37]^
Patients with ASBO	Randomized controlled trial	Open versus Laparoscopic Surgery	Laparoscopic adhesiolysis offers faster recovery in some patients with ASBO compared to open adhesiolysis	Sallinen et al 2019^[38]^
Patients with ASBO	Retrospective study	Open versus Laparoscopic Surgery	Laparoscopic surgery for suspected appendicitis resulted in significantly lower hospitalization rates for ASBO compared to open surgery, though SBO was rare in both groups.	Isaksson et al 2014^[39]^
Patients with ASBO	Randomized controlled trial	Icodextrin 4% solution	The use of 4% icodextrin solution in ASBO is safe and helps reduce intra-abdominal adhesion formation and the risk of re-obstruction	Catena et al 2012^[40]^
Patients with ASBO	Randomized controlled trial	Seprafilm	The use of seprafilm significantly reduced the need for reoperation due to ASBO, and it was the only factor that predicted this outcome	Fazio et al 2006^[41]^

ASBO = adhesive small bowel obstruction.

### 4.1. Conservative management

#### 4.1.1. Nutritional support

In the context of ASBO management, particularly for individuals awaiting surgical intervention or those under conservative care, the provision of adequate nutritional support is imperative.^[21,22]^ Initial management often necessitates a period of Nil Per os to alleviate intestinal pressure and mitigate the risk of further obstruction.^[21,22]^ During this phase, parenteral nutrition is administered to fulfill the nutritional requirements of the patient until gastrointestinal function recuperates sufficiently to allow for the gradual reintroduction of oral or enteral feeding (Table 1).

#### 4.1.2. Intestinal decompression

The technique of intestinal decompression, executed through the insertion of a nasogastric tube, aims to ameliorate symptoms by reducing the accumulation of gases and fluids within the gastrointestinal tract, thereby facilitating an attempt to reestablish gut patency.^[22–25]^ This modality has demonstrated efficacy in a subset of patients with mild to moderate obstructions, notably in cases devoid of ischemic or necrotic complications (Table 1).

#### 4.1.3. Pharmacological intervention

Pharmacotherapy in ASBO primarily targets symptom relief and the prevention of secondary complications. Recent advancements have introduced the utilization of prokinetic agents, such as Metoclopramide and Naloxone, to enhance gastrointestinal motility.^[24,26–36]^ Furthermore, the judicious application of antibiotics has been recognized for its potential in curtailing bacterial overgrowth and translocation, particularly in patients predisposed to infectious complications (Table 1).

### 4.2. Surgical intervention

#### 4.2.1. Indications and optimal timing

Surgical intervention is typically reserved for patients who exhibit refractory responses to conservative management or those who develop severe complications, including ischemia, necrosis, or perforation of the bowel.^[37]^ The judicious determination of surgical timing is paramount to averting adverse outcomes and optimizing patient survival. Premature surgical endeavors may introduce unwarranted risks, whereas delays can precipitate condition exacerbation (Table 1).

#### 4.2.2. Conventional surgical approaches

Conventional surgical techniques generally entail laparotomy, during which adhesive formations responsible for the obstruction are meticulously excised, and, if necessary, compromised intestinal segments are resected.^[28–31,35]^ Despite its efficacy, this approach is associated with significant recuperative demands due to its invasive nature (Table 1).

#### 4.2.3. Minimally invasive techniques

The advent of minimally invasive surgery has significantly altered the therapeutic landscape for ASBO, with laparoscopic interventions offering a less invasive alternative characterized by diminished recovery periods and reduced postoperative adhesion formation.^[38,39]^ Empirical evidence suggests that laparoscopy, when applied to appropriately selected patient cohorts, effectively addresses obstruction while concurrently mitigating the risk of subsequent adhesive complications (Table 1).

#### 4.2.4. Postoperative adhesion prevention

The prophylaxis of postsurgical adhesions remains a pivotal component of ASBO management.^[40,41]^ Strategies include the implementation of meticulous surgical techniques to minimize tissue trauma, the application of barrier agents (e.g., hyaluronic acid, carboxymethylcellulose) to prevent tissue adhesion, and the promotion of early postoperative mobilization and intestinal function recovery to decrease adhesion risk^[40,41]^ (Table 1).

In essence, the management of ASBO necessitates a tailored approach, predicated on a thorough assessment of patient-specific factors, to realize optimal therapeutic outcomes.

## 5. Emerging therapeutic approaches for ASBO

The evolution of medical science has ushered in a plethora of novel therapeutic strategies in the management of ASBO. These cutting-edge approaches not only focus on the direct resolution of ASBO but also emphasize preemptive measures against adhesion formation, aiming to diminish obstruction recurrence and elevate patient quality of life.

### 5.1. Biomaterials for adhesion prevention

In the aftermath of ASBO or abdominal surgical interventions, the deployment of specific biomaterials as physical barriers emerges as an efficacious strategy to thwart adhesion formation.^[42,43]^ Conventional options, such as hyaluronic acid and carboxymethylcellulose-based films or gels, act as physical barriers by isolating injured tissues during healing.^[42,43]^ These materials are intrinsically biodegradable, eliminating the need for secondary removal and therapy improving postoperative recovery while reducing complication rates.

Recent advancements have expanded this paradigm with electrospun nanofibrous matrices, which offer enhanced functionality through therapeutic agents incorporation (e.g., antifibrotic drugs or lubricative coatings). Unlike passive barriers, these matrices combine mechanical separation with controlled drug release to actively modulate fibrotic pathways. For instance, lecithin-based nanofibers exhibit both stability and lubricity, significantly reducing abdominal adhesions.^[44]^ Similarly, drug-eluting nanofibrous scaffolds inhibit collagen deposition and inflammation, minimizing tissue adhesions.^[45]^ Such innovations highlight the potential of multifunctional biomaterials to improve surgical outcomes.

### 5.2. Innovations in drug delivery systems

Innovations in pharmacological delivery, specifically drug coatings, and controlled-release systems, stand as a formidable arsenal against adhesion formation.^[42,46]^ Implemented during surgical procedures, these systems methodically administer anti-inflammatory or antifibrotic agents directly to areas prone to adhesion, effectively minimizing their development.^[42]^ An exemplar application includes bioabsorbable films imbued with anti-inflammatory agents, which significantly attenuate the inflammatory milieu post-surgery, thus reducing adhesion risk.^[42–44]^

### 5.3. Advancements in endoscopic techniques

The advent of enhanced endoscopic technologies has significantly recalibrated the therapeutic landscape for ASBO management.^[7]^ These minimally invasive techniques allow for the alleviation of obstructions sans extensive surgical incisions, markedly reducing patient recovery timelines and the spectrum of postoperative complications.^[7]^ Furthermore, endoscopic adhesiolysis is employed for the management of incipient or mild adhesive conditions, curtailing the reliance on conventional open surgery.

### 5.4. Frontiers in immunomodulation and molecular interventions

The frontier of immunomodulatory and molecular interventions opens new vistas for ASBO management by targeting key molecular pathways in adhesion formation.^[47]^ Current strategies focus on 2 principal mechanism: modulation of fibrotic signaling cascades and direct genetic/epigenetic interventions.

Targeted therapies against fibroblast growth factors and transforming growth factor-beta (TGF-β) are being actively investigated for their capacity to interrupt the inflammatory and fibrotic cascades driving adhesion formation. Particularly promising is the role of microRNA-29b (miR-29b), which suppresses fibrosis by inhibiting TGF-β1/Smad3-mediated collagen synthesis, as demonstrated in pulmonary, renal, and peritoneal fibrosis models.^[48–50]^ Preclinical studies show that therapeutic delivery of miR-29b nanoparticles significantly reduces adhesion severity in ASBO models.

At the cutting edge, CRISPR-based technologies are revolutionizing adhesion prevention. While CRISPR/Cas9 enables precise gene editing, the CRISPR-dCas9 system offers a non-DNA-modifying alternative that epigenetically silences profibrotic genes, achieving > 70% adhesion reduction in murine models.^[51]^ These approaches - combining cytokine modulation with miRNA replacement and epigenetic editing - represent a multifaceted strategy against fibrosis.

These advancements delineate a progressive trajectory in ASBO management, transitioning from broad anti-inflammatory approaches to targeted molecular interventions. As these technologies mature, they promise to enable personalized therapeutic strategies that are both more efficacious and safer, ultimately improving patient outcomes through minimally invasive, mechanism-driven treatments.

## 6. Challenges in treatment and directions for future research on ASBO

The management of ASBO confronts several hurdles despite the availability of a diverse array of therapeutic interventions. These challenges are not solely confined to the refinement of treatment outcomes but extend to encompass patient management and the pursuit of novel research avenues.

### 6.1. Significance of multidisciplinary teams

The efficacy of ASBO management is significantly augmented by the integrated efforts of a multidisciplinary team, comprising surgeons, gastroenterologists, radiologists, nutritionists, and nursing staff.^[10]^ This collaborative model facilitates a holistic evaluation and management of the patient’s condition, ensuring the development of tailored treatment strategies. The synergy within such a team enhances patient outcomes, minimizes the duration of hospital stays, and reduces the likelihood of recurrence.

### 6.2. Patient management challenges

Management of ASBO patients is fraught with challenges, notably the risk of recurrence and the development of chronic complications.^[1,15]^ Despite preventative strategies, postoperative recurrence of adhesions and obstructions remains prevalent, adversely affecting patient quality of life and mental health. Additionally, chronic complications arising from obstruction, including intestinal damage, malnutrition, and electrolyte imbalance, demand persistent and detailed attention.

### 6.3. Prospects for future research and therapeutic innovation

Recent advances in artificial intelligence and precision medicine are transforming the management of ASBO. Machine learning algorithms now enable dynamic risk stratification by integrating clinical variables (e.g., prior surgical history and inflammatory markers), radiomic features from abdominal CT scans,^[52]^ and genomic biomarkers such as TGF-β1 polymorphisms. Predictive models like the random forest approach have demonstrated particular efficacy in high-risk populations, including infants with intestinal malrotation.^[53]^

Concurrently, regenerative medicine approaches are showing promise, with human placental stem cell therapies significantly reducing adhesion formation in preclinical trials.^[54]^ Looking ahead, the convergence of these technologies may enable personalized prevention strategies, including patient-specific 3D-bioprinted peritoneal grafts seeded with mesenchymal stem cells, which have shown an 85% reduction in adhesions in animal models. These innovations collectively represent a paradigm shift toward precision medicine in ASBO management, combining advanced diagnostics with targeted therapeutic interventions.

To summarize, while progress has been achieved in the treatment of ASBO, the journey is far from complete. Future endeavors should continue to evolve and innovate upon the current treatment paradigm to improve outcomes, mitigate recurrence and complications, and ultimately elevate the quality of life for affected individuals. The adoption of an interdisciplinary approach, precision medicine, and advanced technological applications heralds a promising future in the management of ASBO, aiming to deliver enhanced care and therapeutic success.

## 7. Summary

In the realm of ASBO management, the medical field has witnessed substantial progress. This progress encompasses the establishment of collaborative, multidisciplinary approaches, the extensive utilization of minimally invasive surgical techniques, and innovative prevention strategies involving biomaterials and drug coatings. These advancements have significantly improved treatment efficacy, diminished the incidence of postoperative complications, and enhanced the overall quality of life for patients. Despite these achievements, challenges persist, including elevated rates of recurrence, the emergence of long-term complications, and the need for more individualized treatment protocols.

As we look ahead, the continuous evolution of molecular biology and bioengineering promises the development of novel therapeutic strategies. These strategies, including immunomodulatory treatments and molecular interventions, aim to further mitigate adhesion formation and reduce the likelihood of recurrence. Concurrently, the integration of artificial intelligence and big data analytics is poised to revolutionize treatment planning, ushering in an era of more targeted and personalized therapeutic regimens. Furthermore, an emphasis on robust patient management and the fostering of interdisciplinary collaborations are deemed essential for amplifying therapeutic success and minimizing adverse outcomes. Anticipated advancements in technology and therapeutic modalities are expected to usher in significant breakthroughs in the management and treatment of ASBO, paving the way for improved patient outcomes and quality of care.

## Author contributions

**Conceptualization:** Chang-Jun Shen, Jin-Jiang Wang.

**Data curation:** Chang-Jun Shen, Jin-Jiang Wang.

**Investigation:** Jin-Jiang Wang.

**Methodology:** Chang-Jun Shen, Jin-Jiang Wang.

**Project administration:** Jin-Jiang Wang.

**Resources:** Chang-Jun Shen, Jin-Jiang Wang.

**Supervision:** Jin-Jiang Wang.

**Validation:** Chang-Jun Shen, Jin-Jiang Wang.

**Visualization:** Chang-Jun Shen, Jin-Jiang Wang.

**Writing – original draft:** Chang-Jun Shen, Jin-Jiang Wang.

**Writing – review & editing:** Chang-Jun Shen, Jin-Jiang Wang.
